# Cross‐cultural Adaptation and Psychometric Evaluation of the Macedonian C‐DEMQOL: A Carer Quality of Life Measure using data from the NOMAD Trial

**DOI:** 10.1002/alz70858_106446

**Published:** 2025-12-26

**Authors:** Andrea Ivanovska, Marija Taneska, Nicolas Farina, Antoni Novotni, Svetlana Iloski, Mia Micevska, Julia Fischer, Vildan Dogan, Milos Milutinovic, Ljubisha Novotni, Shpresa Hasani, Timo Grimmer, Alexander Kurz, Vesna Dimitrova, Ivan Chorbev, Boban Joksimoski, Gabriela Novotni

**Affiliations:** ^1^ Institute for Alzheimer's Disease and Neuroscience, Skopje, n.a., Macedonia, The former Yugoslav Republic of; ^2^ University of Ljubljana, Ljubljana, n.a., Slovenia; ^3^ University of Plymouth, Plymouth, United Kingdom; ^4^ University Clinic of Psychiatry, Skopje, n.a., Macedonia, The former Yugoslav Republic of; ^5^ Faculty of Medicine, Ss. Cyril and Methodius University, Skopje, n.a., Macedonia, The former Yugoslav Republic of; ^6^ Center for Cognitive Disorders, Technical University of Munich, School of Medicine and Health, Munich, Bavaria, Germany; ^7^ Faculty of Computer Science and Engineering, Ss. Cyril and Methodius University, Skopje, n.a., Macedonia, The former Yugoslav Republic of; ^8^ University Clinic of Neurology, Skopje, n.a., Macedonia, The former Yugoslav Republic of

## Abstract

**Background:**

Caregivers of people with dementia face reduced quality of life, especially in Low‐ and Middle‐Income Countries, due to various systemic, social, and cultural challenges. Accurate measurement of their quality of life is essential for shaping policies and evaluating interventions. In North Macedonia, over 30,000 people with dementia rely mainly on family caregivers, who receive little to no support, resulting in significant stress and burden. However, no validated tools currently exist to measure caregivers’ quality of life. This study aimed to assess the psychometric properties of C‐DEMQOL, a reliable and valid tool for evaluating caregiver quality of life, in a North Macedonian context.

**Method:**

The C‐DEMQOL was back‐translated, and three cognitive interviews with caregivers were conducted to confirm cultural relevance and face validity. Then it was administered to 120 dyads of individuals with dementia and their caregivers as part of the NOMAD (North Macedonia Interprofessional Dementia Care) trial. The internal consistency (Omega) was reported for the entire C‐DEMQOL measure and each subscale. Data were limited to baseline responses. Intraclass correlation was used to estimate the test‐retest reliability of control data by comparing baseline and follow‐up measurements. Convergent validity was assessed through correlations with the Patient Health Questionnaire (PHQ) and the Zarit Burden Inventory (ZBI), while discriminant validity was evaluated by examining correlations with the age and gender of the person with dementia.

**Result:**

C‐DEMQOL demonstrated excellent internal consistency, with four of the five subdomains showing acceptable to excellent internal consistency. The total C‐DEMQOL score also demonstrated good test‐retest reliability, and most subdomains reached acceptable levels. Only the “Feeling supported” subdomain showed weaker internal consistency and test‐retest reliability. Convergent validity was indicated by moderate to large negative correlations with the PHQ and ZBI, while near‐zero correlations with the person with dementia's age and gender supported discriminant validity.

**Conclusion:**

The Macedonian C‐DEMQOL is the first caregiver quality‐of‐life measure in Macedonian to demonstrate satisfactory validity and reliability. Further qualitative and quantitative analyses could help enhance the psychometric properties and optimize the final measure. Validating the C‐DEMQOL paves the way for accurately assessing the effectiveness of initiatives aimed at improving caregivers' quality of life.

